# Patient-specific risk factors for adverse outcomes following geriatric proximal femur fractures

**DOI:** 10.1007/s00068-022-01953-8

**Published:** 2022-03-24

**Authors:** Nils Becker, Tobias Hafner, Miguel Pishnamaz, Frank Hildebrand, Philipp Kobbe

**Affiliations:** Department of Orthopedics, Trauma and Reconstructive Surgery, RWTH Aachen University Hospital, Aachen, Germany

**Keywords:** Geriatric trauma, Proximal femur fracture, Risk factors, Complications

## Abstract

**Background:**

Proximal femur fractures (PFFs) occur frequently among geriatric patients due to diverse risk factors, such as a lower bone mineral density and the increased risk of falls.

**Methods:**

In this review, we focus on recent literature of patient-specific risk factors and their impact on common complications and outcome parameters in patients with PFF.

**Results:**

Patient- and treatment related factors have a significant impact on outcome and are associated with an increased risk of mortality, impairments in functional rehabilitation and complicative courses.

**Conclusion:**

Geriatric patients at high risk for complications are nursing home inhabitants suffering from severe osteoporosis, dementia and sarcopenia. The early and ongoing assessment for these individual risk factors is crucial. Strategies including interdisciplinary approaches, addressing comorbidities and facilitating an optimal risk factor evaluation result in a beneficial outcome. The ongoing ambulant assessment and therapy of complicating factors (e.g., malnutrition, sarcopenia, frailty or osteoporosis) have to be improved.

## Introduction

Proximal femur fractures are common injuries in elderly patients. Based on the anatomic classification, PFFs are subdivided into fractures of the femoral head, the femoral neck, and intertrochanteric or subtrochanteric fractures. For the specific fracture region, several suitable surgical treatment options are described and include arthroplasty and external or internal fixation (such as intramedullary nailing or sliding hip screws). The optimal surgical strategy has been reviewed extensively elsewhere and is determined based on international guidelines [1, 2]. In addition to the fracture patterns, this strategy also has to consider the patients’ comorbidities, prognosis and potential risk factors.

The etiology of PFF differs between younger patients, who usually suffer high-impact injuries, and older patients, who usually have lower bone mineral density and suffer low-impact injuries such as falls from standing height [3]. The vast majority of cases are seen in elderly patients, which is also reflected by an age-dependent increase in the annual incidence of PFFs. White et al. examined a British population and found that the majority of cases occur in the age group of 85–89 years, with an annual age-related incidence of 2237 per 100,000 inhabitants [4]. Taking the increasing life expectancy into account, the total number of PFFs is estimated to increase tremendously within the next decades. In 1992, Cooper et al. predicted a total global number of hip fractures of 3.94 million in 2025 and 6.26 million in 2050 [5]. More recent studies come to similar results, with a predicted increase of total numbers by 36.7% until 2031 [4].

In addition to the individual consequences, this development also brings a relevant socioeconomic burden to the health care systems caused by the direct costs of hospitalization, surgery and rehabilitation. Additional costs result from impaired functional recovery and decreased mobility, which can lead to the need for long-term care [6].

In addition to the enhanced total number of PFFs in the aging population, an associated increase in patients with significant comorbidities is expected, thus complicating the peri- and postoperative treatment of geriatric patients. More than 95% of all patients with PFF present with at least one comorbidity, while the majority of patients have two or three comorbidities. Despite hypertension as the most common comorbidity, anemia, fluid and electrolyte disorders and chronic pulmonary diseases are seen in over 20% of all patients presenting with PFF [7]. Furthermore, distinct comorbidities are known to negatively affect 30-day mortality after PFF, including dementia, cardiac disease, chronic obstructive pulmonary disease (COPD) and renal dysfunction [8]. This becomes even more relevant when looking at the overall outcomes of the large group of patients with PFF. Up to 7% of these patients are affected by early death within 30 days after the fracture [9], and 20–28% face death within the first year after the injury [10]. In addition to the high mortality rates, it must be considered that a high percentage of patients suffering from PFF are not able to continue their lives as independently as before the trauma, leading to serious changes within daily life for the majority of the injured patients once the acute phase passes. This is reflected by a significantly reduced quality of life (QOL) after a PFF [10]. One key factor influencing QOL in geriatric patients is the living situation. In this context, approximately one out of six patients who live at a home-dwelling location before the fracture need to permanently move to a nursing home, resulting in a significantly reduced QOL [11, 12].

The main factors leading to fractures in elderly individuals are the elevated risk of falling and the increased incidence of osteoporosis [13]. Therefore, preventive measures focused on these risk factors should be optimized to make the best use of their potential. In addition to treatment options for osteoporosis, strategies to reduce the risk of falls are therefore of upmost importance. The increasing fall risk of patients over the age of 65 years is of multifactorial genesis. Reduced physical resources, declining cognitive capacities, sensorimotor and sight disorders combined with an environment unsuitable to elderly individuals result in the prevalence of annual falls ranging from 28 to 35% among individuals aged 65 and above [14]. Various assessments are available for identifying individuals at risk of falling. These tools focus on their current living situation. Tests for hospitalized patients differ from tests predicting the hazard of patients in a community-dwelling setting [14]. Approaches to reduce falls in geriatric patients were analyzed in a meta-analysis including 159 trials [15]. A significant reduction in the risk of fall and fall-related fractures was achieved by using multiple component groups or home-based exercise, including balance and functional training, strength training, 3D training (such as Tai Chi) or general physical activity. Especially in patients with sight disorders and a higher risk of falls, home safety assessments were sufficient to reduce the rate of falls [15]. Furthermore, programs including these strategies were shown to have the potential to reduce the resulting costs for the health care system [16].

Despite the necessary efforts to prevent PFF, the total number of PFF in elderly individuals is nevertheless predicted to increase. Thus, the importance of adequate management of geriatric patients will further increase within the coming decades. The assessment of risk factors leading toward a compromised clinical outcome is, therefore, essential to reduce mortality and morbidity by optimized treatment concepts.

## Risk factors

### Frailty

Frailty, defined as the reduced physiologic capacity of geriatric patients to react to an acute stressor affecting several organ systems, is a major risk factor for both the occurrence of PFF and a complicated clinical course after PFF. Therefore, the presence of frailty should be assessed in every geriatric patient [17–19]. The individual nature of frailty makes the diagnosis difficult and is one reason for the lack of an international unified classification. Recent methods to identify frailty are often only reliable in a specific setting, such as primary care, hospital care, or long-term care [19]. Here, we concentrate on studies including patients suffering an acute fracture.

The clinical frailty scale (CFS) is a baseline category grading system that classifies patients over the age of 65, ranking from very fit to severely frail [20], focusing on the clinical impression and the need for help in daily life before the acute injury. Although potentially biased by the examining clinician [21], it can be assessed easily and rapidly. When classified in a higher category of frailty, the patient’s mortality rate is significantly increased following PFF [22–25]. Low et al. found that a higher CFS category is associated with poorer recovery of mobility, lower functional recovery and lower return rates to a community-dwelling setting [26].

In contrast to the clinical assessment by the CFS, the modified frailty index (mFI) concentrates on 19 preexisting deficits, e.g., impaired cognition, history of falls or syncope, thyroid disease, depression or renal diseases [27]. When patients with PFF present with an mFI of 4 or greater, the odds ratio for 1-year mortality is 4.97, as described by Patel et al. [27]. This correlation of the mFI with mortality after PFF has been confirmed by others [28, 29]. Inoue et al. also demonstrated a correlation of an elevated mFI with the occurrence of postoperative complications such as delirium, deep thrombophlebitis, pneumonia, urinary tract infection and pressure sores, lower rates of functional recovery and the new need for institutional care taking [30]. In addition to the CFS and the mFI, an additional way of initially assessing frailty is the usage of simple scores, such as the Identification of Seniors at Risk (ISAR) score [31], which was designed in the setting of an emergency department and offers the opportunity to detect endangered patients at an early stage. Other less frequently used diagnostic frailty instruments describe additional correlations between a higher level of frailty and fracture-associated mortality, complications, reduced activity of the daily life (ADL), longer hospital stay, and a reduced QOL [17, 32–36]. Thus, independent from the method used to assess the level of frailty, the majority of the reviewed studies describe frailty as an independent risk factor for post-fracture mortality and an adverse outcome.

However, despite the relevance of frailty, the therapeutic options are limited. The main aspects of the management of frailty include slowing the progression of physical frailty, optimizing the management of comorbidities, improving the remaining organ function and their medication, strengthening intrinsic capacities such as vision and hearing, assessing psychosocial resources, individually discussing possible outcomes and the patient’s will, and reducing the risk of falls [19]. These interdisciplinary strategies are already implemented in modern approaches to orthopedic care in the perioperative setting for patients with PFF and include regular interdisciplinary consultation and revision of patient-specific factors in a geriatric trauma section.

### Living situation

The living situation before the fracture has an impact on the prevalence of PFF and the patient’s outcome. Patients with the need for care have a higher risk of developing a PFF than patients who are independent in a community-dwelling living situation. Patients with a need for care (home dwelling or at nursing homes) account for up to 50% of patients with PFF [37–39]. Furthermore, the hazard ratio for mortality of patients who were living in nursing homes before the injury is 1.8 compared to patients not living in such an institution [40]. The prefracture resident status also affects the functional recovery within the first year. Thus, institutionalized patients showed lower improvements in their functional and cognitive tests at the 12-month follow-up [41].

### Comorbidities

#### Osteoporosis

Osteoporosis is one of the main factors for the increasing incidence of PFF in geriatric patients and is defined as reduced bone mass with an impaired microstructure [42]. The diagnosis is made by assessing the bone mineral density by dual energy X-ray absorptiometry (DXA) or quantitative computed tomography (QCT). New approaches include bone turnover markers to evaluate the therapeutic response [43] or genetic analysis [44]. Among elderly individuals, osteoporosis is particularly prevalent in postmenopausal women, mainly due to estrogen deficiency [45]. However, long-term corticoid treatment and systemic diseases affecting bone formation and turnover (such as diabetes, chronic kidney diseases, multiple myeloma, primary hyperparathyroidism or immobilization) are also relevant factors leading to osteoporosis [46].

The prevalence of concomitant osteoporosis in patients with PFF varies in the recent literature but can reach 74.9% [47], making the PFF an indicator for manifest osteoporosis. Therefore, when patients are admitted with a PFF, screening for osteoporosis in the perioperative setting should become a standard procedure, as it can greatly improve the rate of diagnosed osteoporosis [48]. The presence of osteoporosis must also be considered when deciding between fracture stabilization or arthroplasty, as demonstrated by Kim et al., who reported an elevated rate of osteosynthesis failure in patients with unstable fractures and osteoporosis who were treated with a dynamic hip screw [49].

It is well known that the diagnosis of osteoporosis is associated with diverse complications after PFF. In this context, an increased fracture-associated mortality has been described [50], which was shown to be attenuated after the prescription of an anti-osteoporotic therapy [51–53]. Furthermore, a significantly enhanced risk for a secondary fracture after a previously undergone osteoporotic fracture as well as a reduction in both hip functional scores and QOL has been reported in cases of osteoporosis and the absence of adequate treatment [54]. Despite these beneficial effects, adherence to anti-osteoporotic therapy decreases in the postoperative phase [55, 56]. Therefore, patients should undergo ongoing screening for their anti-osteoporotic therapy at every follow-up examination.

The basic anti-osteoporotic treatment consists mainly of two groups of drugs, bisphosphonates and vitamin D plus calcium, which can be complemented or exchanged by denosumab, parathyroid hormone and parathyroid hormone-related protein analogs, selective estrogen receptor modulators, menopausal hormone therapy and tibolone or calcitonin, depending on the patients’ characteristics [57]. Many studies highlight the preventive effects of anti-osteoporotic drugs on fracture risk in postmenopausal women [58], while recent studies also describe beneficial effects of bisphosphonates and vitamin D and calcium in men with osteoporosis [59].

Bisphosphonates belong to a group of antiresorptive drugs that reduce the activity of osteoclasts and the associated remodeling rate, thereby inducing secondary mineralization of the bone and increasing bone mineral density [60]. Adverse events from bisphosphonate usage include the risk of atypical femur fractures, which is particularly present in cases of an intake of this type of medication for longer than 5 years. However, the benefits of bisphosphonates in the prevention of osteoporotic fractures greatly outweigh the risk of atypical femur fracture [61]. The usage of the bisphosphonate risedronate in osteoporotic women was associated with a relative risk for hip fractures of 0.54 by reducing the rate of PFF in a 3-year follow-up from 7.4% in placebo-treated patients to 3.4% in risedronate-treated patients [62].

In contrast, the effects of vitamin D and calcium for the treatment of osteoporosis and the associated effect on the fracture risk are not that clear for all subgroups of patients. Beneficial effects were described by Tang et al. in a meta-analysis, reporting a reduced overall fracture risk by 12% and a reduced hip bone loss of 0.54% in patients over 50 years of age using more than 1200 mg calcium plus 800 international units (IE) of vitamin D per day [63]. The reduced risk for PFF was confirmed by other studied that reported a 16% decrease in the risk of PFF in patients with a combined daily intake compared to patients with no therapy or placebo. This significant reduction was not seen in patients using monotherapy with vitamin D or calcium alone [64, 65]. However, the reduced risk for PFF by the aforementioned combined therapy was not confirmed in a specific sub-analysis of the high-risk group of community-dwelling geriatric patients [66], consistent with findings of other trials describing benefits, especially in institutionalized elderly individuals [67].

More studies are needed to investigate the optimal subpopulation of patients benefiting from therapy with calcium and vitamin D. It has to be assessed whether the standard recommendation for postmenopausal women [67] is also beneficial in other subpopulations. These considerations should also respect the possible side effects, as the intake of calcium with or without vitamin D can increase the risk of both cardiovascular insults [68] and lithiasis [64].

#### Cognitive disorders

Aside from the general reduction in cognitive capacities, mental disorders complicate the treatment of geriatric patients with PFF. Distinct neurologic diseases are known to increase the risk of refracturing after PFF [50]. In particular, dementia and delirium have been described to be associated with adverse outcomes in geriatric patients with PFF.

A preexisting dementia in patients with a PFF is significantly associated with a diminished rate of patients returning back in their community-dwelling homes, an impaired recovery of mobility to a prefracture level and increased rates of readmissions to hospital compared to patients with no known history of dementia [26, 69–71]. A diagnosed dementia was also identified as an independent risk factor for 6-month mortality following PFF [72]. Thus, special considerations must be taken to improve the situation of hospitalized patients with dementia.

Regarding the treatment of demented subpopulations with PFF, there is low-quality evidence of a benefit for patients treated in an interdisciplinary manner in a geriatric ward [73]. However, high-quality trials that address the treatment of the growing population of these patients are missing. The current general concepts mainly focus on educational training for caregivers [74], while these strategies must be continuously analyzed and adapted with respect to the predicted increasing incidences within the coming decades. In particular, since patients with dementia are prone to developing complicative delirium, an optimized treatment of dementia has the potential to reduce rates of delirium as well [75–77].

Delirium is defined as acute and fluctuant disturbance in attention and awareness [78] and is common in geriatric patients who suffer an acute injury. The incidence of postoperative delirium in patients with PFF ranges from 17 to up to 50% [79, 80]. In these patients, delirium is a serious risk factor for an adverse outcome. Its development correlated with an impaired recovery of mobility, reduced functional recovery [26], discharge into a nursing home and an increased one-year mortality [77]. Moreover, the early detection of delirium is challenging, particularly in patients with mental disorders. Therefore, a rough initial assessment can be performed by short and easy assessment tools such as the Confusion Assessment Method for the Intensive Care Unit (CAM-ICU) score [81].

While the underlying treatment options are limited, efforts to prevent and recognize delirium early are key factors when treating geriatric patients. The recognition of potentially treatable triggers is crucial and should be routinely realized. These triggers include sepsis, hypoglycemia, stroke, liver failure, dehydration, pain, psychological stress, overdosed delirogenic drugs or day–night disorientation [82]. Measurements addressing these triggers, including reorientation, assisted and monitored fluid intake, aided sitting and walking, optimized pain management, medication and reduced opioid use, have been shown to effectively reduce the risk of delirium. These promising strategies might help to reduce the incidence of delirium, a complication representing an additional psychological burden for patients, relatives and caregivers [78, 80].

### Nutrition

Malnutrition is an additional independent risk factor for adverse postoperative outcomes in patients with PFF. Guimeiro et al. found an association between malnutrition assessed by the Mini Nutritional Assessment (MNA) and increased mortality after 6 months [83]. This association has been confirmed in other studies [84, 85]. Furthermore, malnutrition negatively affects the ADL, rates of postoperative complications, length of hospital stay, mobility, and readmission rates [17]. The reported prevalence of malnutrition in patients with PFF varies widely, mostly depending on the survey method used. Helminen et al. reported a prevalence of 7% for concomitant malnutrition [86], whereas Inoue et al. calculated 25% in a comparable study [87]. There are numerous diagnostic assessments available, although the MNA has been the most widely used [88]. Despite knowledge of the negative impact of malnutrition on the clinical outcome, the beneficial effects of nutritional interventions for patients with PFF are controversial. In a systematic Cochrane Review, Avenell et al. found only low-quality evidence for the effects of nutritional therapy on mortality and very low-quality evidence regarding the development of complications. These findings also include increased protein intake as well as supplementation of iron, amino acids, minerals, vitamin D and other vitamins [89]. However, the ongoing compliant intake of oral supplements and an adequate protein level were associated with lower rates of postoperative complications, such as a reduced rate of pressure ulcers [90]. Therefore, screening for malnutrition should be performed not only during hospitalization but also regularly by the general practitioner to initiate an early and therefore more effective therapy.

### Sarcopenia

Sarcopenia is one of the independent, yet often underdiagnosed, comorbidities influencing the postoperative outcome. Several studies have demonstrated significant negative outcomes after PFF associated with sarcopenia, including reduced ADL and a reduced QOL [91, 92]. Moreover, a significant association between sarcopenia and increased mortality has been demonstrated in a systematic meta-analysis [93]. Kim et al. observed a significantly elevated 5-year mortality of 82.7% among patients with sarcopenia and PFF compared to 52.7% among patients without additive sarcopenia [94].

Data about the incidence of sarcopenia vary between 11 and 76.4% of the overall population with PFF [17], emphasizing the relevance of sarcopenia in this vulnerable population [95]. The syndrome itself is characterized by progressive deterioration of skeletal muscle mass and function. The widely accepted and 2018 revised criteria and cutoff values of the European Working Group on Sarcopenia in Older People (EWGSOP2) define sarcopenia by a reduced grip strength (male < 26 kg, female < 16 kg) and a reduced muscle mass (appendicular skeletal muscle mass divided by height in square; for male patients below 7 kg/m^2^, for female patients below 5.5 kg/m^2^) [96]. To assess the general muscle mass, the usage of dual energy X-ray absorptiometry (DXA) represents the current gold standard. Alternatively, cross-sectional computed tomography (CT), magnetic resonance imaging (MRI) or bioelectrical impedance analysis (BIA) with adjustments for age, ethnicity, and hydration status can be performed [96]. By assessing physical performance (e.g., gait speed), sarcopenia can be further subdivided by its severity. Despite the known negative impact of sarcopenia on the clinical outcome, sarcopenia remains severely underdiagnosed and is rarely considered in the clinical routine [97]. A possible explanation for this might be that to date, there are no approved pharmacologic therapies for sarcopenia due to inadequate efficacy or severe side effects [97]. Once the diagnosis is made, the treatment is predominantly limited to an increase in physical activity [98]. The effect of nutritional supplementation, especially increased protein intake, is controversial [99]. However, some research groups endorse a multimodal approach of a combined nutritional intervention with rehabilitation exercise [100].

The multiple adverse short- and long-term outcomes emphasize the need for sufficient sarcopenia screening and early consistent treatment in the elderly population.

### Selection of treatment

Additionally, international guidelines suggest an operation, in patients with a limited overall prognosis and severe comorbidities as well, to improve pain control and enable mobilization. Therefore, surgical treatment is performed in 95% of all patients with PFF. Hence, large comparative trials on nonsurgical treatment are lacking, leaving only retrospective approaches. A Dutch metanalysis assessed 2615 patients with nonoperative treatment. In two-thirds of these cases, the surgery was denied due to unfit patients with low pretraumatic function; in the remaining cases, the surgery was denied due to nonmedical reasons (such as the patient or their relatives refusing surgical treatment). The outcome of conservatively treated patients was significantly worse with respect to overall mortality and recovery [101]. Thus, the decision for a conservative treatment should only be made in exceptional cases.

When focusing on the surgical therapy of PFF, patients’ comorbidities and overall status as well as fracture morphology are important factors for the choice of treatment option (e.g., arthroplasty or osteosynthesis). Aside from complications such as infections or osteosynthesis failure, various complications have been reported to occur due to technical mistakes, e.g., malalignment, malrotation or elongation of the femur. Potential operative complications have been extensively reviewed elsewhere and should be carefully considered when determining the optimal surgical strategy [1, 102, 103].

### Time to surgery

A well-described and widely accepted risk factor highly affecting patient mortality and the overall outcome is the time from admission until surgical treatment. Most studies have described that a delay between injury and surgery of 48 h is associated with an increased risk for an adverse outcome [9, 104–107]. A more rapid approach was analyzed by Fu et al., who described further benefits on mortality for patients receiving surgical treatment within 24 h after admission [108]. In addition to mortality, surgery within 24 h correlated with a reduced rate of wound infections compared to delayed surgery [109]. Interestingly, no additional significant differences in mortality or the development of major complications were found by a recent study comparing an even faster approach with surgery within six hours after admission compared to 24 h [110]. However, this study also included patients under the age of 65, while patients receiving their surgery within 48 h were not included [110, 111]. Taken together, more studies are needed to assess the optimal time point of operative treatment in general. Despite the mentioned considerations, special attention should be devoted to the individual’s comorbidities, as the beneficial effects of early surgery on outcomes are not consistent for all patients. In this context, some authors demand a preoperative optimization of comorbidities, as distinct disease patterns and the associated overall status of the patient can lead to increased mortality in patients undergoing early surgery [112]. It is of upmost importance to identify subpopulations that can benefit from delayed surgery, as the majority of trials describe a clear general advantage in the majority of patients undergoing early fracture stabilization [104, 113, 114].

## Conclusion

The reduced physiologic capacities in elderly individuals lead to an increased risk of falling. Often, these low-impact falls result in PFF induced by osteoporosis and other comorbidities. Geriatric patients suffering a PFF are at high risk to have a complicative course. Patients at high risk for complications are nursing home inhabitants suffering from severe osteoporosis, dementia and sarcopenia. The early and ongoing assessment for these individual risk factors is crucial and should be a standard procedure in the care of geriatric patients. Various strategies have already been implemented, including interdisciplinary approaches, thus addressing comorbidities and facilitating an optimal risk factor evaluation and resulting in a beneficial outcome. It must also be emphasized that the ongoing ambulant assessment and therapy of many complicating factors (e.g., malnutrition, sarcopenia, frailty or osteoporosis) have to be improved, as the long-term adherence to therapeutic approaches is low.
